# The preventive effect of *Zingiber officinale* essential oil on demyelination of corpus callosum in a cuprizone rat model of multiple sclerosis

**DOI:** 10.22038/AJP.2023.22784

**Published:** 2023

**Authors:** Valiollah Moradi, Seyed Mostafa Ghanadian, Bahman Rashidi, Nazem Ghasemi, Gholamreza Dashti, Ebrahim Esfandiari

**Affiliations:** 1 *Department of Anatomical Science, School of Medicine, Isfahan University of Medical Sciences, Isfahan, Iran *; 2 *Department of Phrmacognosy, School of Medicine, Isfahan University of Medical Sciences, Isfahan, Iran*

**Keywords:** MS, Zinger, Rat, Prevention

## Abstract

**Objective:**

Multiple sclerosis (MS) is the most prevalent neurological disability among young adults. Anti-inflammatory drugs have shown to be effective in MS. The anti-inflammatory and antioxidative properties of *Zingiber officinale* (ginger) have been shown and proven in many phytotherapy studies. This study aimed to evaluate effects of ginger essential oil on preventing myelin degradation in a rat model of MS.

**Materials and Methods:**

In this study, we divided 49 rats into 7 groups; 4 control and 3 experimental groups that received 3 different dose of ginger essential oil (50, 100, and 150 mg/kg/day) for treatment of cuprizone-induced demyelinated rats. Basket test and transmission electron microscopy were performed in this study. *Olig2* and *Mbp* genes and proteins were respectively evaluated by reverse transcription-polymerase chain reaction (RT-PCR) and enzyme-linked immunosorbent assay (ELISA).

**Results:**

Histologically, cuprizone created demyelination in the corpus callosum fibers. Remyelination of fibers was seen in the group treated with the medium dose of ginger essence, by toluidine blue staining. transmission electron microscopy (TEM) revealed increased thickness of the myelin of fibers in all 3 treated groups (p<0.05). Feeding by the medium dose of ginger essence significantly increased the levels of *Mbp* and *Olig*2 genes (p<0.05). ELISA test showed that 100 mg/kg/day of ginger caused a significant difference between experimental and the cuprizone-induced groups (p<0.05).

**Conclusion:**

Our findings suggested that administration of ginger essential oil prevented demyelination and improved remyelination of rats` corpus callusom and can be used as an effective substance in the prevention of MS.

## Introduction

Multiple Sclerosis (MS) is an autoimmune disorder that affects the protective sheath of fibers in the central nervous system (CNS). It is the most common chronic inflammatory disease of the CNS in young adults (Oberwahrenbrock et al., 2013 ▶; Ransohoff et al., 2015 ▶; Sinnecker et al., 2012 ▶). Demyelination and early neurodegeneration are two distinct characteristics of MS (Milo and Miller, 2014 ▶). This abnormal condition can develop into a complex pattern of physical or cognitive disability, and neurological defects (Ghasemi et al., 2017 ▶; Shakerin et al., 2020 ▶). Demyelination of axon myelin sheath and destruction of the axons within the CNS are some of the characteristics of this disease (Milo and Miller, 2014 ▶). 

The exact cause of MS is not known, but, genetic factors and environmental exposures can be introduced as a reason (Poppe et al., 2008 ▶). 

There has been no decisive treatment for MS yet. MS therapeutic attempts have improved the neuronal function and stopped the disease progress (Compston and Coles, 2002 ▶). The use of herbal medicines in MS patients has increased dramatically over the past decades (Dayapoglu and Tan, 2016 ▶; Kim et al., 2018 ▶). Treatment with medicinal herbs has been shown useful for treating various disorders since ancient times (Mikaili et al., 2013 ▶; Shakerin et al., 2020 ▶). Previous studies have suggested that herbal remedies have many therapeutic effects on various disorders, including cancer, diabetes, and neurogenic diseases (Agyare et al., 2018 ▶; Reisi et al., 2014 ▶). 

Ginger or *Zingiber officinale* Roscoe (Zingiberaceae) is a routinely used spice. Also, it has been recommended for the treatment of various illnesses in traditional Asian medicine (Wattanathorn et al., 2011 ▶), such as stomach ache (Mascolo et al., 1989 ▶), nausea and diarrhea, and joint and muscle pain (Ojewole, 2006 ▶). The ginger extract also has antioxidant (Ahmed et al., 2005 ▶; Kuo et al., 2005 ▶; Masuda et al., 2004 ▶; Nanjundaiah et al., 2011 ▶; Stoilova et al., 2007 ▶), neuroprotective (Waggas, 2009 ▶), and anxiolytic (Hasenöhrl et al., 1996 ▶) effects. It contains over 400 different types of compounds; however, the greatest healing effect of ginger is due to its gingerols, shogaols, zingerone, and paradols (Prasad and Tyagi, 2015 ▶). Ginger and some of its derivatives can be used to treat some autoimmune disorders, such as MS.

The effect of three extract of fresh ginger,6-gingerol,8-gingerolo and 10-gingerol on murine T lymphocytes, were reported to be potent inhibitors of T lymphocytes suggesting that ginger could be beneficial in chronic inflammatory conditions associated with excessive or inappropriate T lymphocyte activation (Bernard et al., 2015 ▶). This effect of ginger was also found in experimental autoimmune encephalomyelitis (EAE) induced mice (Jafarzadeh et al., 2014 ▶). Therefore, ginger can suppress some immune responses, such as antigen presentation, T cell activation, and IFN-γ and interleukin-2 secretion by T cells (Tripathi et al., 2008 ▶). 

Besides, studies have shown an improvement in the severity of disease symptoms and a decrease in immune and inflammatory changes in ginger-treated EAE mice (Jafarzadeh et al., 2017 ▶). Ginger potentially prevents leukocyte penetration into the CNS of EAE mice (Jafarzadeh et al., 2014 ▶). 

Some studies have shown the protective effects of ginger against some neurodegenerative diseases, including epilepsy, migraine, Alzheimer's and Parkinson's disease (Choi et al., 2018 ▶). 

In previous researches, the anti-inflammatory and regenerative effects of ginger have been proven to some extent, but so far, no detailed study showed the effect of ginger essential oil on the process of remyelination of nerve fibers. This study aimed at assessing the efficacy of ginger essential oil on preventing myelin degradation in a rat model of MS induced by cuprizone. We investigated this issue by behavioral, histological and molecular tests. We used *Mbp* and *Olig*2 genes, while the former is an important gene in myelination of nervous system fibers, the latter is a marker for oligodendrocyte cells.

## Materials and Methods

**Plant materials**

**Preparation of the essential oil**

The ginger pieces were bought from a market store in Isfahan (Iran). Ginger was characterized and approved by the pharmacognosy department of Pharmacy School in Isfahan University of Medical Sciences. Then, 4 kg of the fresh ginger plant rhizome was air-dried in the shade (500 g), powdered, and underwent hydro distillation using a Clevenger-type apparatus for 3 hrs. (each time 100 g of the powder and for 5 times) according to the method recommended by the British Pharmacopoeia (Pharmacopoeia, 1988). The obtained volatile oils were mixed, dried over anhydrous sodium sulfate, and stored in a sealed vial at 4°C until analysis.

**Gas chromatography-mass spectrometry (GC-MS)**

The analysis of essential oil was done on Agilent gas chromatograph, Model 7890A, equipped with a 5975C mass detector and a triple quadrupole mass analyzer and electron ionization (EI). The gas chromatograph was equipped with an HP-5 capillary column (30 m×0.25 mm; film thickness: 0.25 μm). The oven temperature was started from 50ºC, held for 2 min, raised by 8ºC/min up to 250ºC, followed by 250-330ºC by 3ºC/min with the total runtime of 58 min. Helium was used at a flow rate of 2 ml/min as the carrier gas. Injector and detector temperature was 280°C. Ionization voltage was 70 eV; ion source temperature was 230°C and mass range was 50-1000 m/z as MS operating conditions. The MSD Chem Station was used as operating software. 

Components of the sample were identified by comparison of their mass spectra and retention times with those reported in relevant studies.

**Animals**

Male 8-week old Wistar rats weighing 200-250 g were provided from Pasteur Institute, Tehran, Iran and placed in groups of 3 animals per cage in standard polypropylene cages at 22±2ºC under 10:14 hr light-dark cycle. All animals had free access to food and water. This research was approved by the Ethical Committee of Isfahan University of Medical Sciences (Ethics code: IR.MUI.REC.1395.3.262)

A total of 49 rats were divided into seven groups as follows: Group I: control group with no intervention (Cont); Group II: MS control group induced by cuprizone that not treated with any treatment (Cup); Group III: Sham group, including the rats with MS that received sodium carboxymethyl cellulose (NaCMC) as Fingolimod and ginger solvent (Sham); Group IV: MS induction+Fingolimod (0.5 mg/kg/day) treatment (Fing); Groups V-VII: Experimental groups that received cuprizone and different doses of ginger essential oil orally (50, 100, and 150 mg/kg/day: EssL, EssM, and EssH, respectively). Treatment with Fingolimod and ginger essential oil was initiated one week before the induction of MS for 4 weeks. 

**Creating a demyelination model**

The demyelination lesion was induced using cuprizone (bis-cyclohexanone oxaldihydrazone, Sigma-Aldrich Inc. C9012). To obtain the desired cuprizone model, we gavaged the rats for three weeks with 0.6% cuprizone, dissolved in corn oil. Three weeks after cuprizone feeding, histological examination of the brain was done to verify the demyelination. Treatments led to oligodendrocyte apoptosis and demyelination of fibers which was examined in the corpus callosum (Ransohoff et al., 2015 ▶; Abe et al., 2015 ▶).

**Behavioral test**

Basket test was used to assess coordination of motor limbs and sensory-motor defects in the studied groups. It was done twice a day to characterize the motor performance condition and muscle resistance of the rats during the study. After training, we placed each rat in the floor of the wire mesh basket (W=25cm×L=40 cm), and then, the basket was suddenly turned upside down. The results were evaluated by scoring the delay to fall in 180 sec (Stamenkovic et al., 2017 ▶).

**Cardiac perfusion and sampling**

After the induction of MS, animals were anesthetized by combination of Ketamine (100 mg/kg) and xylene (10 mg/kg). The chest was opened, and perfused transcardially with the normal saline, followed by 4% formaldehyde (Merck, 1040021000) (pH 7.4). The skull was dissected and the brains tissue were removed, post fixed in the fixative solution for 24 hr then 5-μm thickness sections were prepared by rotary microtome (Leitz1512, Germany), stained with toluidine blue and examined using an Olympus Provis optical microscope (Behnam et al., 2000 ▶).

**Transmission electron microscopy**

Rats were transcardially perfused with 1.6% glutaraldehyde (Sigma-Aldrich G5882) in phosphate-buffered saline (PBS) (0.12 M, pH 7.4). The corpus callosum was detached and fixed by 1% osmium tetroxide (Sigma-Aldrich, 75632) and embedded in epoxy resin after dehydration in graded ethanol. After embedding in resin, ultrathin 70-nm sections were prepared and stained with uranyl acetate and lead citrate and observed using a transmission electron microscopy (TEM) (LEO 906 Germany, 100 kV). Images were taken from corpus callosum after removing from rat brain. Pictures of the fibers cut in cross-section were taken and myelin status was assessed using ten images per specimen (×3000) analyzed by Digimizer Image Analysis Software 5.3.5 (copyright © 2005-2019 MedCalc software). The percentage of myelinated axons, axon diameter, myelin thickness, and G-ratio were measured using 50 fibers per sample. The G-ratio was calculated by the axon diameter/entire fiber diameter ratio. Hence, a completely demyelinated fiber has a G-ratio of 1, whereas in the myelinated fibers, the G-ratio is <1 (Hedayatpour et al., 2013 ▶).

**Total RNA isolation and quantitative RT-PCR**

This method was used to evaluate the expression of oligodendrocyte transcription factor (*Olig*2) and myelin basic protein (*Mbp)* genes in CC. Total RNA was extracted using the BioFACT™ Total RNA Prep Kit (BIOFACT™, Korea) based on the instruction sheet. The DNA of the samples was removed from the extracted RNA, by treating them with the RNase-free DNase kit (Qiagen, Germany). RNA quantity and quality were determined by using spectrophotometry at 260 nm and 280 nm. Primers used in this study were designed by AlleleID 7.6 and checked against the rat genome using the BLAST site. Gene expression was normalized against *Gapdh* as a housekeeping gene. All primer sequences for *Gapdh*, *Olig*2, and *Mbp* are listed in Table 1.

In the next step, complementary DNA (cDNA) was synthesized using the BioFact™ RT-Kit (BioFact™, Korea). Real-time PCR was carried out by BIOFACT™ 2X Real-Time PCR Master Mix (High ROX) using the specifically mentioned primers and performed on a Step One Plus system (Applied Biosystems). The comparative expression level of intended genes was calculated using the 2^- ΔΔct^ method (Razavi et al., 2013 ▶).

**Table 1:** Sequences of primers used for each gene

Gene	Forward Primer	Reverse Primer	Size (bp)
*Gapdh*	*ATGACTCTACCCACGGCAAG*	*GGAAGATGGTGATGGGTTTC*	87
*Olig*2	*CACAGGAGGAACCGTGTCCT*	*GGTGCTGGAGGAAGATGACT*	145
*Mbp*	*TCACAGAAGAGACCCTCACAGC*	*GAGTCAAGGATGCCCGTGTC*	116

**Enzyme-linked immunosorbent assay (ELISA) for Olig2** **and** **MBP protein**

The concentration of Olig2 and MBP protein in CC of rat brain tissues was measured by the standard ELISA method.Rats’ CC was extracted and weighed followed by homogenization in PBS containing protease inhibitors using sonication (Bertin Technology, France). Homogenates of CC were centrifuged (11000 rpm for 20 min at 4°C) and the supernatant was extracted. The assays were performed based on the manufacturer's protocol. Finally, duplicate plates were read on an ELISA reader at 540 nm (Guan et al., 2011 ▶). 

**Statistical analysis**

The results of the evaluation techniques were determined as mean±standard error of the mean (S.E.M). Data analysis was performed using SPSS 24 software and the one-way analysis of variance (Stoilova et al.), followed by the LSD post-hoc test. A p<0.05 was defined for statistical significance.

## Results

**GC–MS Standardization of the sample**

**Figure 1:**
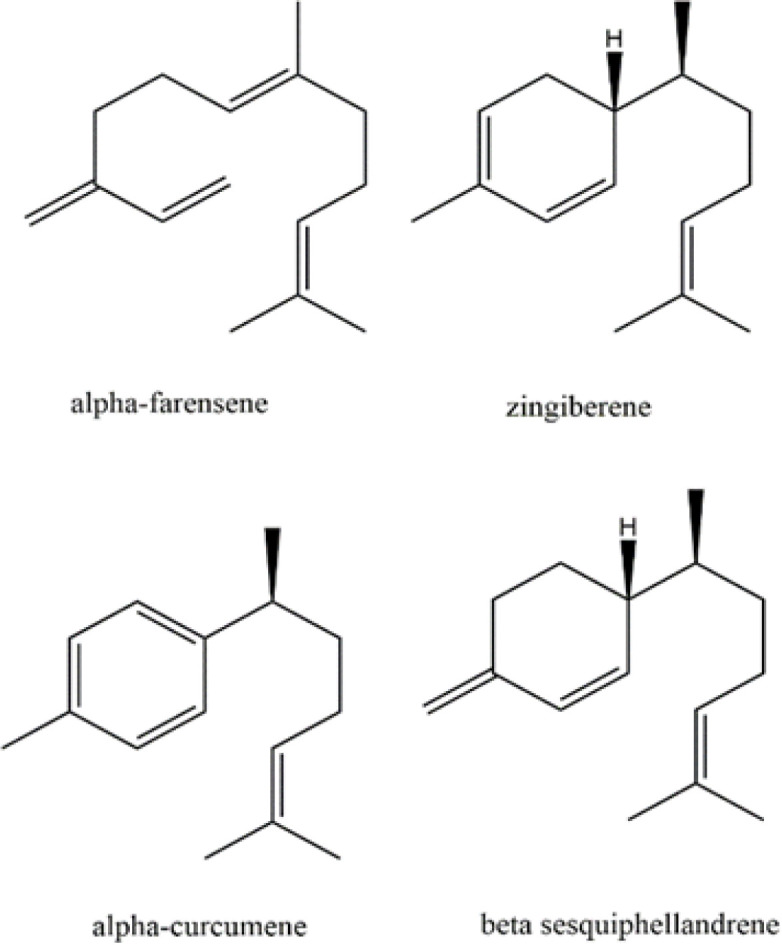
Farnesene derivatives in ginger oil

Based on GC-Mass spectra and total ion count (TIC) chromatogram of the sample, it was standardized on its major components related to sesquiterpene farnesene derivatives (29.11‬% TIC), including alpha-curcumin (6.21%) at a retention time of 17.6, zingiberene (9.55%) at a retention

 time of 18.3, alpha- farnesene (6 to 6.47%) at a retention time of 18.8, and beta-sesquiphellandrene (6.88%) at a retention time of 19.2 min (Figure 1).

**Behavioral test**

**Figure 2:**
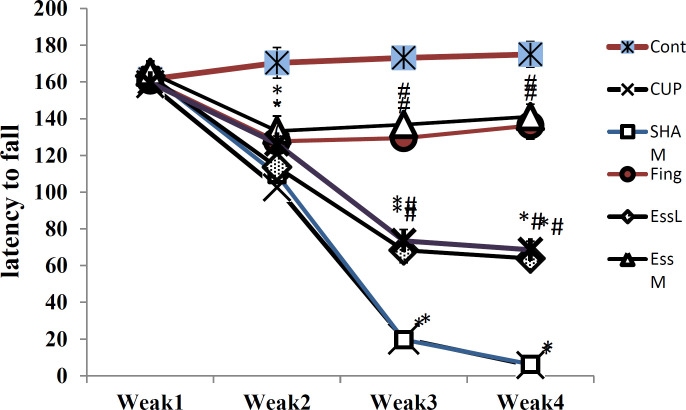
Basket test for the evaluation of rats behavior in the experimental groups. The figure depicts that the latency to fall time significantly reduced in all the cuprizone groups compared to control group (p<0.05). In groups treated with fingolimod and the medium dose of *Zingiber officinale* (ginger) essential oil, the latency significantly increased (p<0.05), whereas there was no difference between the fingolimod and the medium dose of ginger essential oil groups and control group. *: Significant difference with the control group. #: Significant difference with the cuprizone and sham groups. Healthy control group (Cont), MS control group (Cup), group received sodium carboxymethyl cellulose (NaCMC) as Fingolimod and ginger solvent (sham), fingolimod group (Fing), *Zingiber officinale* (ginger) essential oil groups (50, 100, and 150 mg/kg/day: EssL, EssM, and EssH, respectively).

Basket test showed no significant differences between all groups in the time of climbing down the wall in the first week. At the end of 4^th^ week, the sham and cuprizone groups had no significant differences; however, based on Figure 2, the latency to fall in the CPZ and sham groups decreased significantly compared to the experimental groups (p<0.05). Also, the fingolimod-treated group and the group treated with the medium dose of ginger essential oil had no significant differences; but there was a significant increase in latency to fall time between these two groups, in comparison to groups that received cuprizone (p<0.05). Whereas, no significant differences were seen between the low and high doses of ginger essential oil (Figure 2). 

**Myelin staining**

**Figure 3:**
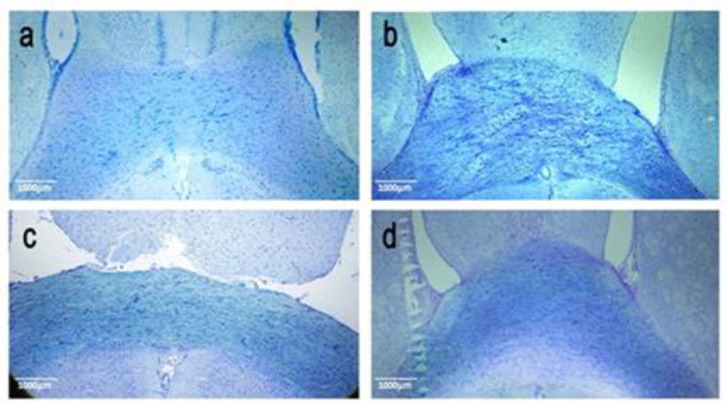
Cross-section of the rats’ corpus callosum showed the demyelination of this region in the cuprizone group. Remyelination improved in treatment groups, especially by fingolimod and the medium dose of ginger (*Zingiber officinale*) essential oil. a: control group (Cont), b: MS control group (Cup), c: Fingolimod (Fing), and d: Medium dose of ginger essential oil

Demyelination of CC fibers was seen after toluidine blue staining in tissue sections of cuprizone groups. The figure 3 highlight, the remyelination in the groups receiving fingolimod and the medium -dose of ginger essential oil with increased significance in comparison to the Cup and sham groups.

**Transmission electron microscopy (TEM)**

**Figure 4:**
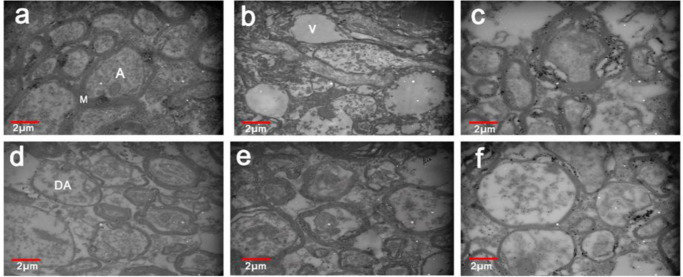
The images obtained by the transmission electron microscope from the corpus callosum fibers of the rats’ brains in the comparing groups. Demyelination in the cuprizone and sham groups, as well as the destruction of the demyelinated axons (Ben-Chaim et al.) and vacuole formation (V) can be seen. In the EssM group, remyelination is done properly (TEM 2000x). a: control group, b: MS control group, c: Fingolimod group, d-f: *Zingiber officinale* (ginger) essential oil groups (50, 100, and 150 mg/kg/day: EssL, EssM, and EssH, respectively)

**Figure 5:**
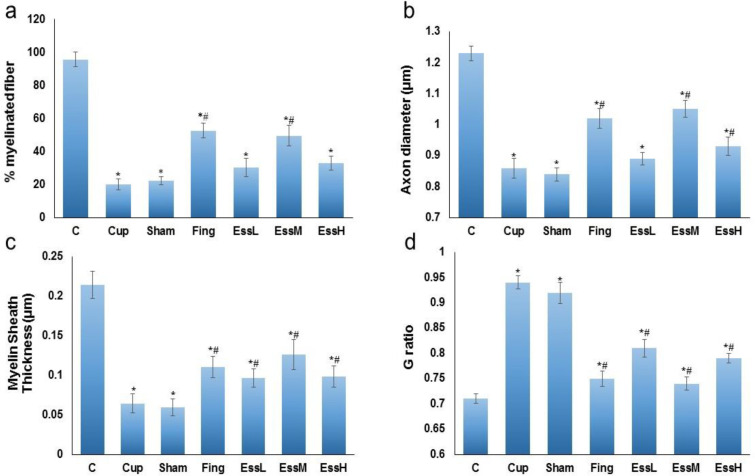
a: The mean±SEM of the percentage of myelinated fiber in the experimental. Fing. and EssM groups showed significant difference compared with the Cup. and sham groups (p<0.05). b: The mean±SEM of axon diameter in the experimental groups. The axon diameter in the Fing. and EssM groups showed significant difference compared with the Cup. and sham groups (p<0.05). c: The mean± SE of the myelin thickness in the experimental groups. Fing. and EssM groups showed significant difference compared with the Cup. and sham groups (p<0.05). d: The mean±SEM of G-ratio in the experimental groups. The G-ratio in the treatment groups, especially in the Fing. and EssM groups significantly reduced in comparison with the Cup. and sham groups (p<0.05). *: Significant difference with the C group. #: Significant difference with the Cup. and sham groups. Control group (C), MS control group (Cup), sham group (sham) Fingolimod group (Fing), *Zingiber officinale* (ginger) essential oil groups (50, 100, and 150 mg/kg/day: essential low dose oil(EssL), essential medium dose oil (EssM), and essential high dose oil (EssH), respectively)

Myelin sheath diameter was measured using TEM to determine myelin morphometric parameters. Photographs of corpus callosum fibers were taken by Electron microscopy and analyzed by Digimizer Image Analysis Software to measure the myelinated axons percentage, axon diameter, thickness of myelin, and G-ratio (Figures 4 and 5). Three weeks after cuprizone feeding beginning, the myelin of CC fibers was degenerated. The mean number of myelinated axons was significantly increased in the Fingolimod and EssM groups (p<0.05) compared with the cuprizone groups. After treatment with fingolimod and ginger essential oil, axon diameter increased compared with the cuprizone and sham groups (p<0.05). Evaluation of the TEM photos revealed an increase in myelin thickness in the CC in the Fingolimod and EssM groups in comparison to cuprizone and sham groups (p<0.05).

Furthermore, the mean G-ratio significantly increased in the cuprizone and sham groups in comparison to the control group. The mean G-ratio had significant reductions in the Fingolimod and EssM groups (p<0.05) as shown in Figure 5.

**The** ***Mbp*** **and** ***Olig*** **2 genes expression in CC**

**Figure 6.**
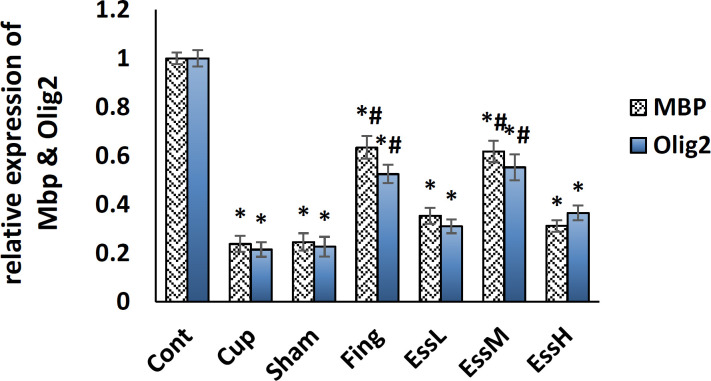
The mean±SEM of the expression levels of *Mbp* and *Olig*2 genes in corpus callosum of the studied groups. The expression levels of *Mbp* and *Olig*2 genes in the ginger essential oil (EssM) and Fing. groups significantly increased compared with other groups (p<0.05). *: Significant difference with the control group. #: Significant difference with the Cup. and sham groups. control group (Cont), MS control group (Cup), sham group (sham) Fingolimod group (Fing), *Zingiber officinale* (ginger) essential oil groups (50, 100, and 150 mg/kg/day: essential low dose oil(EssL), essential medium dose oil (EssM), and essential high dose oil (EssH), respectively)

There was a significant difference between the control and other groups in the expression level of *Mbp* and *Olig2* genes. There was no significant difference between the sham and cuprizone groups. The medium dose of ginger essential oil and fingolimod showed no significant difference; however, they significantly (p<0.05), increased *Mbp* and *Olig2* genes expression levels compared with the cuprizone group’s. The low and high doses of ginger essential oil did not show significant difference (p<0.05); however, they showed a significant reduction compare to medium dose of ginger essential oil and fingolimod (p<0.05) (Figure 6). 

**ELISA results for MBP and Olig2 proteins level in CC**

**Figure 7:**
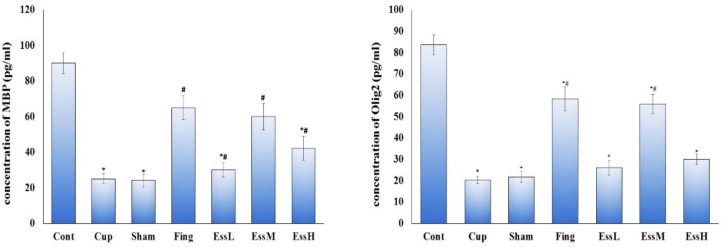
a: The mean±SEM concentration of MBP protein in studied groups, showed no significant difference between Fing.and the medium dose of ginger essential oil groups,. Cup. and sham groups showed significant difference (p<0.05). b: The mean±SEM concentration of Olig2 protein in studied groups, showed no significant difference between Fing. and the medium dose of ginger essential oil groups; but, a significant increase was observed in comparison to Cup. and sham groups (p<0.05). *: Significant difference with the control group. #: Significant difference with the Cup. and sham groups. control group (Cont), MS control group (Cup), sham group (sham) Fingolimod group (Fing), *Zingiber officinale* (ginger) essential oil groups (50, 100, and 150 mg/kg/day: essential low dose oil(EssL), essential medium dose oil (EssM), and essential high dose oil (EssH), respectively)

The protein levels of MBP and Olig2 in all treatment groups were significantly (p<0.05) more than the cuprizone and sham groups. Among the treatment groups, the dose of 100 mg/kg of ginger essential oil showed similar effect like fingolimod group, whereas, the high and low doses of that was observed to have no such therapeutic effect. Treatment with fingolimod and the middle dose of ginger essential oil caused the MBP and Olig2 proteins levels to reach the level close to that of the normal (control) level (Figure 7).

## Discussion

In this study, the effects of *Zingiber officinale* essential oil on the remyelination of corpus callosum induced by cuprizon in a rat MS model, were evaluated. Our result implied that the medium dose of ginger essential oil could improve remyelination and oligodendrocyte regeneration when compared to fingolimode. Several therapeutic methods have been applied by the Food and Drug Administration to use in relapsing-remitting multiple sclerosis (RRMS) and secondary progressive multiple sclerosis (SPMS) through the past decade, those are not effective for primary progressive MS (PPMS) and progressively have become ineffectual for SPMS, particularly in the degenerative stage of the disease. Hence, replacement therapies with more effectiveness should be applied for progressive forms of MS (Hawker, 2011 ▶).

MS is a chronic inflammatory disease, caused by demyelinating of the CNS (the loss of myelin-forming oligodendrocytes) that usually pursued by remyelination, the spontaneous and efficient regenerative process (Minagar et al., 2006 ▶). Up to now, the etiology of this disease has remained unclear and except symptomatic treatment, there is no treatment available (Rolak 2003 ▶; Sarkar et al., 2017 ▶).

Currently, there is no definite cure for MS, therefore, finding a fully effective and safe treatment has been widely considered (Mojaverrostami et al., 2018 ▶). Some drugs such as fingolimod are used in clinic. The reason for choosing fingolimod as a standard drug is that it has been shown to cross the blood-brain barrier using radiolabeling, and this drug can prevent demyelination of axon fibers, and inhibit lymphocyte proliferation, and it has anti-inflammatory properties (Hunter et al., 2016 ▶). Similar to the function of fingolomod, the essential oil of ginger was reported to have the properties of the blood-brain barrier action, (Wattanathorn et al., 2010 ▶) , anti-inflammatory and antioxidant properties (Aryaeian and Tavakkoli, 2015 ▶) and in general its neuroprotective properties (Waggas, 2009 ▶).

Numerous studies have shown that fingolimod and ginger are both able to cross the blood-brain barrier (Hunter et al., 2016 ▶) and both have anti-inflammatory and neuroprotective properties that can inhibit myelin degradation (Salvati and Di Biase, 2014 ▶). So far, no known side effects have been reported for ginger, while fingolimod has many side effects, including high blood pressure, skin necrosis, hair loss, etc. (Behjati M et al., 2014 ▶)

Ginger can be presented as an alternative and complementary approach to potentially improve brain disorders due to its neuroprotective effects. MS patients have been advised to take ginger because of its anti-inflammatory effects (Jafarzadeh et al., 2014 ▶).

In this study, the MS model was induced by a 0.6% cuprizone-containing diet and for substantiation of the induced MS, marked damages to axons and the myelin sheath were assessed by using TEM. In this study, the results showed that the medium dose of ginger essential oil was more effective than its low dose. All three doses of the ginger essential oil improved all studied variables. In addition, the medium dose of ginger essential oil caused the MBP and Olig2 proteins levels back to the normal level. According to the results, it was found that the medium dose of ginger essential oil had a better effect than low and high doses, in a way that there was a significant difference from the cuprizone and sham groups. 

In relation to drugs that have neuroprotective properties, we can refer to a research, in which the *Rosa damascena* extract proved to be effective in treatment of Alzheimer's disease (Esfandiary et al., 2015 ▶). In our study, medium-dose ginger essential oil had the best effect.

In several studies the effects of ginger on the CNS have been demonstrated, whereas, no similar study found to be done on MS. Definite effects of ginger and its active components (6-gingerol and 10-gingerol) due to their anti-inflammatory and neuroprotective effects have been approved in animal models of MS (Bernard et al., 2015 ▶); however, to verify these results clinical studies are still needed on MS patients (Mojaverrostami et al., 2018 ▶). A study, demonstrated the protective effects of ginger on focal cerebral ischemia in a rat model of brain damage. Moreover, ginger showed reduced cognitive deﬁcits induced by focal cerebral ischemia (Wattanathorn et al., 2011 ▶). Previous studies, reported the effectiveness of ginger in diabetic-induced CNS damage (El-Akabawy and El-Kholy, 2014 ▶). Mehdizadeh et al. (2012) ▶, after induction of neurotoxicity by 3,4-methylenedioxymethamphetamine (MDMA) showed that cell number increased after ginger treatment in comparison with the MDMA group. Also, the down-regulation of Bcl-2 and up-regulation of Bax were observed in the ginger group (Mehdizadeh et al., 2012 ▶). It was revealed that ginger compounds, such as 6-paradol effectively protects the brain after cerebral ischemia possibly by attenuating neuroinflammation (Gaire et al., 2015 ▶). The present study also showed the useful effect of zingiber essential oil in the treatment of anti-inflammatory disturbances in MS rat disease model. Mahboubi in a review of ginger essential oil in 2019 explained its antimicrobial, antioxidant, anti-inflammatory, analgesic, anticancer, and immuno-modulatory effects. Our results also found the anti-inflammatory and immuno-modulatory effects of ginger essential oil by the improvement of remyelination in cuprizone-induced demyelinated CC.

Uniquely, Zhornitsky et al. (2013) ▶, studied the effects of quetiapine fumarate postinjury in a model of “chronic demyelination mice which were fed cuprizone. The results showed that quetiapine blocks cuprizone-induced demyelination and increases remyelination after cuprizone is withdrawn. Similarly, in our study,the ginger oil showed to possessed significant antioxidant properties by blocking cuprizone induced demyelination and increasing remyelination in the animal model. 

In conclusion, our findings suggested that *Zingiber officinale* essential oil administration prevented demyelination and improved remyelination in the CNS. Thus, the essential oil of *Z. officinale *may be used as a preventive and therapeutic agent in some neurodegenerative diseases, such as MS.

## Conflicts of interest

The authors have declared that there is no conflict of interest.
